# Everybody's Page

**Published:** 1890-01-11

**Authors:** 


### Everybody's Page.

Very few people die absolutely of old age. It is very often a prevailing epidemic which carries off the old and feeble.

The colour Prussian blue was discovered accidentally. It it made by mixing impure potassium carbonate with refuse animal matter, such as parings from horses' hoofs.

If any of our readers want to encourage birds to come into their garden, they should try hanging up a raw meat bone on a stick in the garden; it is considered a great delicacy by the feathered tribes.

It is alleged, with what truth we do not know, that one man in every twenty meets with an accident yearly. A few weeks of such weather as we have experienced recently, with roadways fit only to skate upon, would probably increase the percentage in an unpleasant manner.

Ground glass can be made by anyone in the following manner: Place a sheet of glass of the size required on a flat board, sprinkle a small quantity of emery on it, and wet it with water; then place another glass over and grind the two together, renewing the emery and water if necessary.

An easy way to make very wholesome black currant lozenges is to put 1lb. of sugar to four quarts of black currants, prepared in the same way as for jelly. Strain through a sieve into a jar, and place the jar in a saucepan of water and let it simmer till it is quite thick, then let it get a little cool; pour out on to dishes or tins that have been well greased. Cut it up into lozenges, and keep shut up in tin boxes with a layer of paper between each row.

Winter time, frosty days, and east winds make many of the faces we meet in the streets look so cold and pitiful, that it is a great temptation to put our hand in our pocket and give a trifle. Indiscriminate charity really amounts to being rather a selfish sort of pleasure; it hurts us to see a pleading face, and so we give our penny or sixpence, as the case may be, and go on our way rejoicing, not having in nine cases out of ten done any good but to the gin shop. Very few genuine cases of poverty are met with in the streets, and the daily papers constantly remind us how many of the wretched little children who wail for coppers are the agents of systematic beggars.

Hungary and Italy, as well as France, Portugal, and Spain, have forbidden the importation of saccharin.

Nicotine extracted from the tobacco leaf, which is useful for the destruction of parasites on plants, has been tried in Russia with success as a remedy for dry rot among sheep.

London Jews are said to suffer less from phthisis than ordinary inhabitants of London, and the reason is in part due to the strict inspection of their meat. Thus tuberculous meat is excluded from their diet.

It has been said that fully half of the diseases we suffer from are caused by sleeping with closed windows. There is probably some exaggeration in this statement, but the fear of night air which some people have is unreasoning. In large towns the comparative absence of smoke at night makes the air purer than it is in the daytime.

A simple remedy for a disease which has caused much trouble on banana plantations in the Fiji Islands, is reported to have been discovered accidentally by the sea encroaching for a short time on a portion of a plantation. The sea water destroyed the diseased stems, in the place of which healthy and most prolific young shoots came up.

The Doctors Poincaré, and Macé, of Nancy, say that they have found living microscopic germs in alimentary preserves; the germs become temporarily inert while they are buried in the boxes or tins, but they soon come to life again when exposed to the air; and the moral of this, to people who eat tinned things, is that the contents of a tin ought to be used at once.

In 1872 the French peasants hatched more than a million ounces of silkworm eggs, but in 1886 less than half that quantity was used. An interesting report in the *Times* tells us that there is no commercial protection at all for the sericulturists, and the manufacturers of late years have made up their silks from inferior qualities of the raw material which came from China, Japan, India, and Italy. Luckily for the poor peasants, people in France are getting disgusted with the wretched stuff sold as "pure silk," and municipal councils, agricultural societies, etc., are doing their best to get protection for the peasants and raise the good material at home.

